# User Experiences of Behavioral and Psychological Change Techniques in a Walking-Based Mobile Exergame: Cross-Sectional Qualitative Study

**DOI:** 10.2196/78776

**Published:** 2026-03-27

**Authors:** Marianna Antoniadou, Helena Orädd, Aseel Berglund, Erik Berglund, Anna Strömberg, Tiny Jaarsma, Leonie Klompstra

**Affiliations:** 1 Department of Health, Medicine and Caring Sciences, Linköping University, Linköping, SE-581 83, Sweden, 46 11363629; 2 Post Graduate School for Integrated Care, Örebro University, Örebro, Sweden; 3 Department of Computer and Information Science, Linköping University, Linköping, Sweden; 4 Department of Cardiology, Linköping University, Linköping, Sweden

**Keywords:** exergames, physical activity, technology, behavioral change techniques, psychological change techniques, qualitative study

## Abstract

**Background:**

Physical activity plays a central role in the course and progression of chronic conditions in older adults. However, individuals within this population tend to have an inactive lifestyle. Exergaming, which is defined as the integration of physical activity with game-based elements, offers a promising approach to promote physical activity in individuals with chronic conditions. Despite its potential, limited evidence exists on how specific game elements influence behavioral and psychological outcomes in this population.

**Objective:**

The aim of this study is to explore the behavioral and psychological change techniques experienced by individuals with chronic conditions using a walking-based mobile exergame, called Heart Farming.

**Methods:**

A cross-sectional qualitative design was used based on a gamification framework, using data from semistructured interviews with 14 participants aged 67 to 92 years who used the Heart Farming exergame for 3 months. Participants with chronic conditions, including heart failure, Parkinson disease, type 2 diabetes, stroke, or rheumatic disease, were recruited from 2 ongoing studies. Data were analyzed using deductive content analysis and presented based on the gamification framework.

**Results:**

The data analysis revealed increased motivation to walk, which was facilitated by game elements such as goals, rewards, feedback, and planning. Participants valued not only the sense of progression and achievement within the game but also the real-world benefits, such as spending time in nature or feeling a sense of community with others. Exergaming was integrated into daily routines by supporting the planning and structuring of daily activities. It was also perceived as enjoyable, especially due to its farming theme and visual design. Behavioral change techniques such as goal setting, feedback, and social support were commonly experienced, whereas focus on past success (as described in the gamification framework) was not used as a technique by the exergame. Psychological techniques, including self-monitoring and stress management, were mentioned. A feeling of discomfort due to playing in public was reported, and participants varied in terms of the levels of digital literacy. Social interaction features were not adequately used, even though some participants created informal support groups to exergame.

**Conclusions:**

This study contributes to the exergaming literature by examining behavioral and psychological change techniques from the perspective of individuals with chronic conditions. Exergaming incorporates a variety of behavioral and psychological change techniques, which were experienced by the participants in various ways. Individuals’ previous knowledge of exergaming, interests, and illness progression were mentioned as factors that can influence their experiences of exergaming. By increasing the understanding of how game elements are experienced and how they influence health-related behavior, the findings of this study may inform the development of exergames that are better tailored to users’ needs. Further research is needed to refine behavioral change techniques and assess condition-specific adaptations to maximize engagement and health outcomes.

## Introduction

### Background

Approximately one-third of adults worldwide are living with at least 1 chronic condition [1]. Being physically active can have a positive impact on the course and progression of different chronic conditions, such as chronic heart failure [2], stroke [3], or Parkinson disease [4]. However, despite the significance of being physically active, individuals with chronic conditions tend to have an inactive lifestyle. This is characterized by low levels of physical activity and sedentary behavior like sitting or lying down [5].

Gamification is a well-known and widely used approach to increase motivation and user engagement by implementing game design elements in nongame contexts [6], including health care and behavioral change [7]. Gamification emphasizes identifiable behavioral and psychological change techniques, such as goal setting, feedback, and self-monitoring, from which psychological and behavioral change can emerge [8]. Although these techniques can be layered onto existing activities or systems, they can also be part of a unified game structure as a *serious game*. Serious games are games with a primary nonentertainment objective, such as promoting learning, health, or behavioral change, that fundamentally shape the design and function of a game [9]. In their book on serious games, Dörner et al [9] introduced the term “characterizing goal” to describe these primary nonentertainment objectives of serious games. When the characterizing goal refers to exercises or other physical activities, the game is typically called an *exergame*.

Exergaming, which combines gameplay with physical activity in real life, is a relatively new approach to increasing physical activity for individuals with chronic conditions. Exergames are serious games for health that can possibly increase motivation to physical activity [10] and improve physical function, balance, exercise capacity, and energy expenditure [2,11,12]. Although many traditional exergames are designed for indoor use in front of a screen, some individuals might prefer outdoor exergames rather than being confined to screen-based play indoors [13-15]. Mobile exergaming is a suitable option for remote physical activity interventions, particularly when in-person meetings with health care professionals are not possible. Smartphones are equipped with built-in technologies, such as accelerometers, cameras, and sensors [16].

Mobile exergames employ a range of technologies to support and motivate physical activity, including indoor, outdoor, and hybrid solutions. GPS is a technology that relies on satellite positioning to track movement over larger areas. GPS is used in location-based exergames that are primarily suited for outdoor use [17,18]. An example of a game that uses this technology is Pokémon Go [19]. However, location-based exergames, including Pokémon Go, have previously been criticized as inappropriate or even dangerous. Pokémon Go has been shown to contribute to accidents caused by players’ inattention to their surroundings while walking or even driving [20,21]. In contrast, augmented reality is a technology that integrates digital content with the real-world environment using cameras and sensors, enabling interactive and context-aware experiences that can be used both indoors and outdoors, while also supporting precise movement and spatial tracking [16].

Exergames use various techniques that aim to achieve behavioral change and can thus be useful in establishing healthy behaviors in individuals with chronic conditions [22]. The combination of key elements (eg, reward, challenge, feedback, information, goals, and planning) with the main aims of rehabilitation programs, such as motor skills learning, long-term retention, and transfer of skills, makes exergaming a valuable tool for health care professionals [23,24]. Although the physiological component (ie, the physical activity) is important for an exergame to be effective, it is also important to keep the psychological attractiveness (ie, the “fun”) of exergames in mind [25,26]. The attractive nature of exergames might enhance individuals’ motivation to engage in physical activity, which may in turn lead to positive outcomes in terms of dose response, adherence to, and long-term effectiveness of the rehabilitation programs. Although exergaming can help distract from unpleasant feelings like pain, there is a negative correlation between perceived exertion and enjoyment [27,28], and it is important to adapt the intensity of the exercise to avoid feelings of overexertion.

There are several previous studies indicating the health benefits of exergaming in individuals with chronic conditions. A recent randomized controlled trial (RCT) on the home-based and tailored exergaming interventions has shown them to be feasible and safe for individuals with movement disorders, with adjustable difficulty levels, real-time feedback, and balance-focused tasks being particularly important for addressing gait and postural control in Parkinson disease [29]. Findings from studies conducted on people with heart failure demonstrate that exergaming, alongside other exercise modalities such as yoga, can positively influence exercise capacity as well as physical and mental health, highlighting the importance of designing exergames that are safe, motivating, and adaptable to varying functional abilities to support sustained participation among older adults with chronic conditions [30]. In addition, the findings of a systematic review show that exergaming can improve quality of life, physical functioning, and psychological well-being among people living with a wide range of chronic conditions [22]. Gamification-based interventions have also been found to enhance self-management, motivation, and long-term engagement in chronic disease care, further supporting the potential of exergames as a complementary tool in chronic condition management [31].

### Study Objectives

Although there is much research to support the potential health benefits of exergaming, there is a gap in evidence regarding the game elements that impact health behavior and how individuals experience them. To fully understand the potential of exergames as a tool to increase physical activity in people with chronic conditions, further exploration of this population’s experiences of exergaming is required. By gaining insight into how gamification techniques are experienced, researchers and developers may be able to better tailor and adapt exergaming interventions to the user’s needs. Therefore, the purpose of this study is to explore the experienced behavioral and psychological change techniques in individuals with heart failure, Parkinson disease, type 2 diabetes, stroke, or rheumatic disease when using a walking-based mobile exergame.

## Methods

### Research Design Overview

This study adopted a qualitative descriptive cross-sectional study design and was conducted within the Heart-eXergame (Heart-eXg) study, which is an international RCT, and the Heart Farming Chronic Conditions (HF-CC) study. The study was reported in accordance with the Journal Article Reporting Standards for Qualitative Research guidelines [32].

### The Gamification Framework

**Figure 1.**
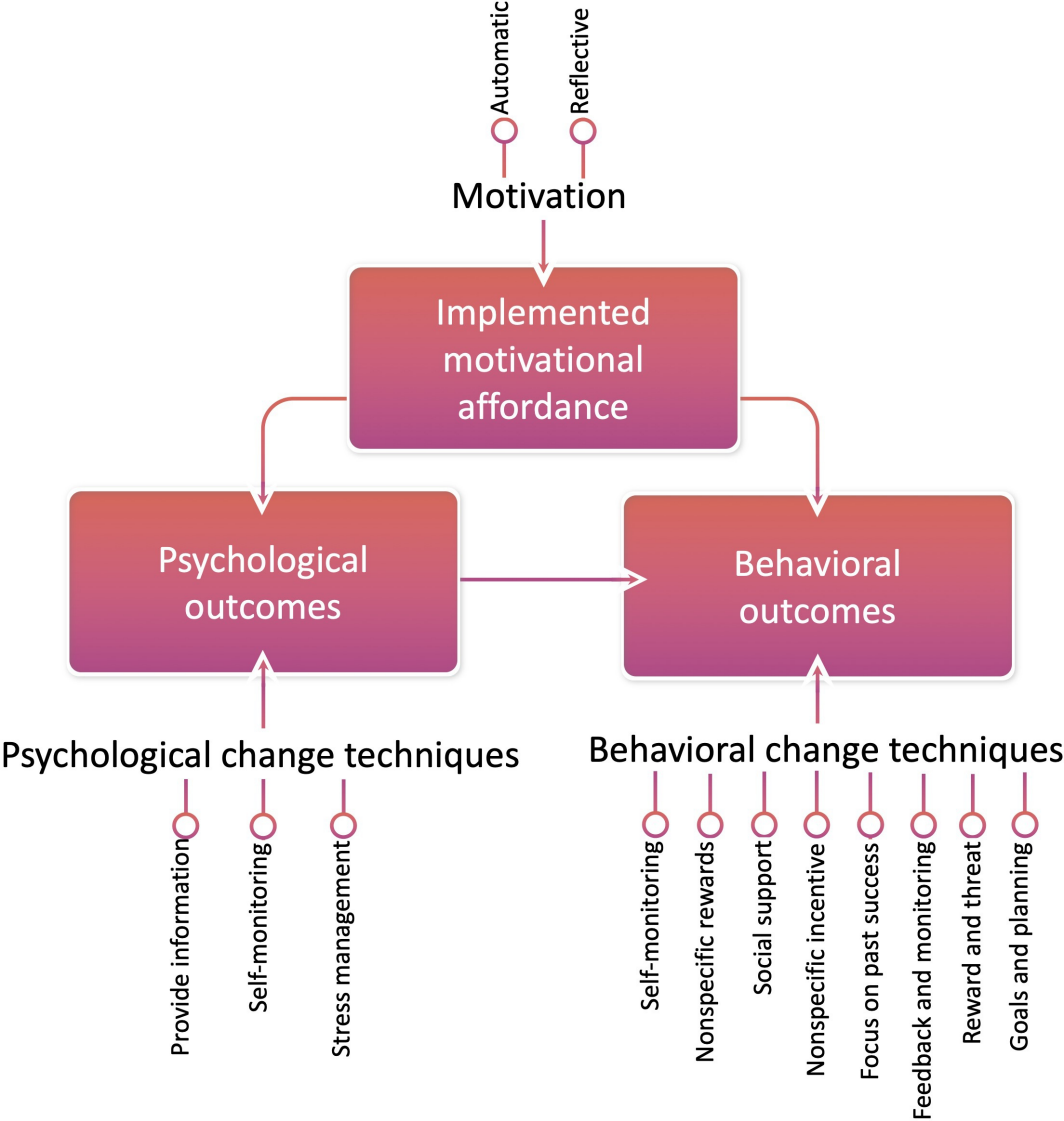
The gamification framework for exergaming by Berglund et al [8], showcasing how behavioral and psychological change techniques influence psychological and behavioral outcomes (reproduced from Berglund et al [8], with permission from the authors under the CC BY-NC 4.0 license).

Gamification may be used in exergaming to maintain behavioral change and encourage physical exercise [33]. Gamification is defined as the use of game elements in a nongaming context, for example, behavioral change [6]. Gamification consists of 3 main components (Figure 1): implemented motivational affordances (properties of the gamified system that allow the user to experience competence, relatedness, and autonomy), psychological outcomes (induced by motivational affordance and psychological change techniques), and behavioral outcomes (induced by motivational affordances, psychological outcomes, and behavioral change techniques) [8]. The psychological change techniques are *providing information*, *self-monitoring*, and *stress management*, while the included behavioral change techniques are *self-monitoring*, *nonspecific rewards*, *social support*, *nonspecific incentives*, *focus on past success*, *feedback and monitoring*, *reward and threat*, and *goals and planning*.

Specific game elements used to impact health behaviors can also be sorted as different principles of gamification [8]. These principles state that the gamified system should provide *meaningful purpose* by aligning goals with the user’s goal or interest, include *meaningful choice* by giving users agency over how they reach the goals, *support player archetypes* by leveraging individual characteristics of the user, clearly communicate *feedback* on how the user’s actions affect their progress, and provide *visibility* over the user’s progress. These principles can be used to sort specific game elements according to their purpose. For example, digital rewards and game levels can be used to communicate progress (visibility) and which actions cause progression (feedback).

### The Heart Farming Exergame

The mobile exergame Heart Farming used in this study was developed by a research team of game developers and health care professionals [16,34]. It was developed using the gamification framework described earlier [8], with the characterizing goal of motivating individuals with heart failure to walk 10 minutes more per day [16].

The exergame uses walking as the main interaction to drive the game forward. To allow for precise tracking both outside and inside, the exergame uses augmented reality technology to track the phone’s, and therefore the player’s, movements by analyzing the view from the mobile phone’s camera [16]. The theme of the exergame is a farm where the player can buy fields and manage them by sowing, watering, and harvesting crops. All actions in the game have a set number of meters the player must walk in real life to complete the action. The distance needed to complete actions is based on the player’s individual walking goals to balance the effort needed for people with different abilities.

To progress in the exergame, the player must create a plan with actions to perform and walk until all tasks are completed. The exergame also included an option called “autoplay,” where a plan is created automatically, allowing the player to walk without actively planning their actions. The created plan will yield random crops and is not as effective in progressing in the game as actively planning. Multiple gamification elements are also used in the exergame as displayed in Table 1.

**Table 1.** Descriptions of key game elements in the Heart Farming walking–based exergame and how they utilize the gamification elements described by Berglund et al [8].

Gamification element	Game elements in Heart Farming
Leaderboards	A leaderboard displays the 3 players who gained the most experience points the previous day.
Levels of achievements or ranks	Levels are based on the player’s experience points, and leveling up will also give access to new game contents.
Score systems	The number of meters the player has walked is used to reach their daily goal, set to how many meters would be needed for that player to walk for 10 minutes.
Experience points reward systems	All actions performed in the game are associated with a number of experience points the player earns when completing them. The amount of experience points earned for an action is the same for all players, even though the meters needed is determined by each player’s own walking ability.
Item-granting system rewards	Players receive a gift each day when they open the exergame. The gift contains a random crop they can sell or store to use later.
Resources	Crops can be sold for money or stored to use for specific achievements, for example, to fulfill requirements for unlocking an animal or a neighbor. The money is used to buy new fields to sow new types of crops and expand the farm and progress in the game.
Achievement systems	Heart Farming has a mission book with animals that can be unlocked and placed on the farm by doing all the tasks specified by that animal. There are also neighboring houses placed on the farm that request certain crops, and selling the requested crops fills up a series of hearts that resets at the start of every week. If all hearts are filled before the end of the week, the player gains a decorative item on that neighbor’s house.
Feedback messages	Sound effects are used to communicate completion of tasks and plans while the player is walking, as well as let the player know if the game is registering walking or not. Animations are used when achieving goals, such as a trophy appearing when reaching the daily goal or a heart expanding to show a new item when completing the requests from a neighbor.
Unlocking mechanisms	The crops the player can grow are limited by their current level and are unlocked when leveling up. Leveling up also unlocks some available actions in the game, such as being able to fish in the river or do squat exercises for extra crops.
Social or peer pressure	Players can send messages to other players in the form of emojis and can help each other by sending crops from their own farm.

### Study Participants

The Heart-eXg study is registered on clinicaltrials.gov NCT05641662. In the Heart-eXg study, participants had to be diagnosed with heart failure by a physician, while participants in the HF-CC study had to self-report at least one of the following chronic conditions: heart failure, Parkinson disease, type 2 diabetes, stroke, or rheumatic disease. In both studies, participants had to be older than 18 years of age and in a stable condition. Participants who were unable to perform a 6-minute walk test or unwilling to use a smartphone to exergame were not included. Further exclusion criteria were severe cognitive dysfunction, anticipated short-term survival, or difficulties in understanding or reading the Swedish language. All participants provided informed consent before participating in the study. Participants in the intervention group of the Heart-eXg study and the participants in the HF-CC study were advised to walk 10 minutes more every day for 3 months using a mobile exergame. For further information, see the Heart-eXg study website [35].

All participants were introduced to the exergame during introductory sessions in the university, outpatient clinics, or offices of the patient organizations, where members of the research team provided oral instructions and assistance to install the exergame on their mobile phones. When needed, the research team offered individual help to participants, including more detailed explanations or providing practical assistance with the exergame. In addition, participants were given step-by-step written instructions (manual) with QR codes to short instructional videos. The manual was provided in Swedish with simple vocabulary and illustrations of the exergame to facilitate the understanding of the different functions/steps in the exergame. During the first month of the intervention, participants also received follow-up calls weekly, where they could also raise questions or issues regarding the exergame. Participants in the intervention group of the Heart-eXg study and the participants in the HF-CC study were all advised to walk 10 minutes more every day for 3 months using a mobile exergame called Heart Farming. Interviews were conducted at the 3-month follow-up.

A purposeful sampling strategy was used to recruit participants with variation in their playing patterns (eg, participants who played extensively and others who primarily used the autoplay function), symptoms, age, and sex. This strategy was chosen to ensure variability within the sample and to capture rich, information-dense data that could illuminate different experiences relevant to the study aim.

### Data Collection

The semistructured interviews were conducted by 3 researchers: one of the authors of this paper (HO), who was actively involved in the exergame’s development as a game developer, and 2 health care professionals (a nurse and an occupational therapist) who were not involved as authors in the study. To mitigate bias, the interviewers did not have any pre-established relationship with study participants. If, for instance, one interviewer conducted follow-up phone calls, another researcher was assigned to perform the interview to maintain objectivity. Participants were not informed of HO’s involvement in the development of the game to minimize the risk of response bias. To further minimize potential bias related to HO’s involvement in developing the exergame, interviews were conducted using a semistructured guide, and reflexive discussions were held within the research team throughout data collection and analysis. In addition, data analysis involved multiple researchers who were not involved in the game’s development, ensuring analytical triangulation and critical appraisal of interpretations.

The interview guide was divided into 3 different components, including the participants’ experiences of playing the Heart Farming exergame, its influence on daily life and habits, and its different game elements. The development of the interview guide was based on the gamification framework developed by Berglund et al [8] to focus on how the gamification elements were experienced by the participants. The interview guide was also informed by Klompstra et al [13] to include the overall experience of using the Heart Farming exergame, including daily life and habits. The interview started with an introductory question (“Can you tell me about your experiences with playing this game for the last 3 months?”) and continued with more specific questions about the exergame. Probing questions, such as “Could you elaborate?” “Could you provide an example?” or “What do you mean by saying that?” were used to facilitate the conversation and provide more accurate descriptions and rich information. A translated version of the interview guide is attached as Multimedia Appendix 1.

A pilot interview was first conducted with a participant to assess the clarity of the questions. After discussions with the research team, no further modifications were needed to the interview guide, based on this pilot interview. Due to the relevance of the information, the pilot interview was included in the data analysis. No field notes were kept by the interviewers. After conducting 12 interviews, information power was achieved because of clear redundancy with repetition of patterns in the data without novel additions [36]. To confirm this, 2 additional interviews were conducted. No additional new information came from the last 2 interviews.

The interviews took place at the participants’ homes, one of the university campuses, or over the telephone, depending on the participants’ preferences and the feasibility of traveling to and from the interview. In 2 phone interviews, the participants’ spouses were present during the interview. During the analysis, the researchers made sure not to include any of the spouses’ comments during the analysis, and only the answers from the participants were included. The interviews were recorded and transcribed for analysis, with the participants’ consent. The length of the interviews varied between 18 and 69 minutes, with an average length of 35 (SD 15) minutes. All interviews were conducted in Swedish, which is the native language of both the participants and the interviewers.

### Data Analysis

The data analysis was performed after the completion of data collection. A deductive content analysis following the steps suggested by Elo and Kyngäs [37] was applied. The framework introduced by Berglund et al [8] was used in a descriptive way to structure the analysis. A categorization matrix was developed based on the behavioral and psychological change techniques introduced in the framework and visualized in Figure 1.

During the preparation phase, all authors read through the transcribed interviews to familiarize themselves with the data and gain an in-depth understanding of the content. In the organization phase, the first 3 interviews were analyzed in parallel by 4 of the authors (MA, HO, LK, and AB), and the meaning units were allocated to the categorization matrix based on the gamification framework for exergaming. Thereafter, the remaining interviews were independently analyzed by the first 2 authors (MA and HO), who equally divided the work. To facilitate the coding process, the NVivo 15 (Lumivero) software was used. Finally, in the reporting phase, the findings were presented in alignment with the structured categories in a way that ensured clarity.

Methodological rigor was ensured by following the guidelines established for qualitative studies, introduced by Guba and Lincoln [38]. Analysis of triangulation was ensured through regular discussions between the authors, aiming at reaching a consensus considering the categorization of the data. The interviews were read multiple times to get an overall understanding of the content, and the parts of the interviews were constantly compared with the whole. Authentic quotations were translated into English and presented as part of the results to increase the trustworthiness of the findings. The translation of the quotations was performed by the 2 first authors (MA and HO), and another researcher (LK) verified the accuracy of the translations.

### Researcher Reflexivity

Two female researchers (MA and HO) conducted the biggest part of the data analysis presented in this paper. Both were PhD students employed at Linköping University. MA had a background in occupational therapy with a master’s education, and HO was a game developer with a master’s education as well. Their personal presumptions were that physical activity can be promoted by the Heart Farming exergame and that it can motivate participants to walk more on a daily basis. Both presumed that exergaming can influence daily life and habits.

Their contribution to developing the interview guide was equal. MA did not participate in data collection, while HO performed the interviews with 2 other health care professionals. The transcription of the interviews and the data analysis were mainly performed by MA and HO. To mitigate biases, part of the data analysis was performed by LK and AB, and a discussion took place considering the data analysis. All the authors gained closeness to the data by repeatedly reading the interview transcripts and listening to the original interview recordings. Reflexive discussions with co-authors AB, EB, AS, TJ, and LK supported MA and HO in enacting researcher reflexivity. AB and EB had a background in game development, AS and TJ had a nursing background, and LK had a background in public health. They were all experienced researchers in the field of exergaming and individuals with chronic conditions.

### Ethical Considerations

The study is registered on clinicaltrials.gov with the ID NCT05641662. The Heart-eXg study and the HF-CC study were conducted in line with the Declaration of Helsinki. The Heart-eXg received ethical approval from the Regional Ethical Review Board in Linköping (Dnr 2021‐03314), as did the HF-CC study (Dnr 2023-06398-01). Participants received written and verbal information about the study’s aim before providing written informed consent to participate.

Participant privacy and confidentiality were carefully protected throughout the study. All collected data were anonymized prior to analysis, and any potentially identifiable information was removed from the transcribed interviews. The anonymized data were stored securely in a separate folder, accessible only to the research team. No financial or other forms of compensation were provided to the participants in this study. The manuscript and all supplementary materials do not include images or other content that could enable the identification of individual participants. As such, additional consent for publication of identifiable materials was not required.

Due to the sensitive nature of personal data and study materials, data cannot be made freely available. However, by contacting the corresponding author, procedures for sharing data, analytic methods, and study materials for reproducing the results or replicating the procedure can be arranged in accordance with Swedish legislation.

## Results

### Participant Details

A total of 14 participants were included in this study. Study participants were individuals with chronic conditions who had access to exergaming in the Heart-eXg or the HFCC study for 3 months. There were 8 (57.1%) women and 6 (42.9%) men with ages ranging from 67 to 92 (mean age 75.3, SD 8.9) years. Five (35.7%) participants were diagnosed with heart failure, 4 (28.6%) with Parkinson disease, 4 (28.6%) with rheumatic disease, and 1 (7.1%) with stroke.

### Deductive Analysis

The data were structured based on the gamification framework by Berglund et al [8], which was partly confirmed by the data from the participants. Regarding the behavioral change techniques, 5 out of 8 could be fully confirmed in the data. The technique “focus on past success” was not used in the design of the exergame, and therefore, participants did not mention it. Participants experienced rewards but not threats as described in “Rewards and threats.” “Nonspecific rewards” was also not used as a technique in the exergame, but participants experienced the social aspects of exergaming, and thus, it is described under social support. “Self-monitoring” could be found but was experienced as psychological rather than behavioral and was thus only included under the psychological change techniques.

### Behavioral Change Techniques

#### Overview

These techniques have been identified as crucial to changing the behavioral outcomes of users in real life. Following the framework, behavioral change techniques include goals and planning, reward and threat, feedback and monitoring, focus on past success, nonspecific incentive, social support, nonspecific reward, and self-monitoring [8]. The exergame used multiple gamification elements, which could be mapped to the techniques according to Table 2. The analysis of the interviews revealed additional experiences connected to behavioral techniques. Although these experiences were not linked to specific gamification elements and are not included in the table, they are discussed further in the text.

**Table 2.** The behavioral change techniques experienced by people with chronic conditions after playing the Heart Farming walking–based exergame for 3 months and the gamification elements facilitating the techniques based on the deductive analysis using the gamification framework by Berglund et al [8].

Behavioral change techniques as described in the framework	Heart Farming gamification elements
Goals and planning	The daily goal, one of the exergame’s *score systems*, gave participants a goal to reach and plan around. This assisted in structuring daily life and developing a gaming strategy.
Reward and threat	The *experience points, reward system,* and a*chievement systems* (eg, collecting animals) worked as rewards that motivated participants to walk more. No threats were mentioned by the participants.
Feedback and monitoring	*Feedback messages* assisted in planning their walk (walking progress) and motivated participants to continue walking to finish their plans (audio feedback).
Focus on past success	Not used as a technique and not mentioned.
Nonspecific incentive	The graphics and farming theme increased motivation.
Social support	The *leaderboard* was used to compare scores with the top 3 players.
Nonspecific rewards	*Social or peer pressure* (via the experience of an exergame community) is described under social support.
Self-monitoring	This was experienced as a psychological, not behavioral, technique.

#### Goals and Planning

Participants described how exergaming assisted in planning and structuring their daily routine. Participants preferred to exergame in the mornings, and they mentioned that they usually have coffee and breakfast directly afterward, as a form of reward for accomplishing their daily goal, which is one of the exergame’s reward systems. Participants also combined their everyday activities such as visiting the doctor or doing grocery shopping with exergaming. Sometimes participants played inside the house, especially when the weather did not allow them to play outside.

*I use it every time I go to the health care center and when I get a blood test, and I also use it when I go to my children and my grandchildren*.

[67-year-old man]

They also mentioned the gaming strategy they used while exergaming and the use of the option to autoplay. Participants who used the game emphasized that they chose to prepare their plan for the game at home, so they did not have to interrupt their walking during the journey. The variety of options within the game provided was appreciated, and participants mentioned that they chose to harvest the farm and collect animals. Autoplay was used when participants wanted a simpler option or they did not want to interrupt their walking.

#### Reward and Threat

Participants mentioned that the digital rewards they received motivated them to walk more. Collecting animals was considered enjoyable, and players chose what actions to take in the exergame according to what was required (eg, harvest 3 fields) to get that animal.

*And then I look at what I am missing, for example, do I need to get a new dog, horse or elephant? Then I work towards gaining them*.

[67-year-old man]

Sometimes participants expressed the need to receive more rewards and money when selling to neighbors and more points when they walked further (after having achieved their daily goal). They mentioned that receiving more money for selling to neighbors might increase their motivation. They felt that if they wanted to walk more daily after achieving their daily goal, that would lead to more rewards.

#### Feedback and Monitoring

The feedback provided from the game assisted the participants in monitoring their behavior. Participants mentioned that knowing how much they walked during the day helped them in planning their walk for the rest of the day.

Participants generally liked the sound and noted that the sound effects helped to understand what happened in the game and made the process more interactive. The footsteps and farming sounds helped confirm if the camera was tracking the distance and that the sounds for reaching a goal or the end of a plan acted as both notification and reward.

*You heard gravel when you walked and it also because a bit of a whip [referencing the expression “carrot and stick,” in Swedish literally “carrot and whip”], and a little carrot as well, to have it there and know that it checks how much you walk*.

[80-year-old woman]

If participants continued exergaming 3 times more after having reached their daily goal, which was 10 minutes of walking daily, the game reminded them to rest. Participants adhered to this suggestion.

#### Nonspecific Incentive

Increased physical activity and perceived improved health were considered motivating factors to continue exergaming. Participants mentioned that they noticed an increase in their walking speed, endurance, and the number of daily steps. Some even mentioned that they noticed weight loss because of increased physical activity, and thus, they felt more motivated to exergame.

I*t was not that I was a big fan of this specific game but measuring the daily physical activity and comparing it with how improved I felt [regarding physical activity level], that was what I liked*.

[77-year-old man]

Participants highlighted that exergaming not only motivated them to walk more but also increased their appetite to go outside and enjoy nature. Exergaming provided them with the “little push,” as mentioned by a 71-year-old woman, and thus, it gave them a purpose to go out and walk with the game, instead of being physically inactive inside.

Participants liked the graphics used in the game. A 68-year-old woman stated that “it’s nice with these fields and the barns in the middle of it all.” They also mentioned that they liked the option provided by the exergame to do squats instead of walking as an alternative that provided some variation. Participants also thought that the graphics were clear and made the game easier to understand, providing an incentive to continue with exergaming. However, participants also mentioned that the graphics were too childish. A 71-year-old man characterized the exergaming as “a game for children.”

Participants generally liked the farming theme of the game. They referred to a sense of nostalgia while exergaming, and specifically, a 68-year-old woman mentioned *“*[It] can be fun to have a farm in your imagination*.”* Participants mentioned that they liked this particular exergame, despite a lack of general interest in games, due to its graphics.

*I don’t have any interest in [games] but this, when I saw that auto-walking man out and walking, that I thought was a little fun, so I was immediately hooked on that*.

[85-year-old man]

#### Social Support

Even though playing with others was an option within the exergame, some participants were not that interested in this function, while others did not have anyone to play with or did not know how to use this function.

Participants were interested in using the leaderboard as a form of competition by comparing their own scores with the top 3 players displayed on the leaderboard. They also suggested ways to make it easier to see your own score compared to those of the other players, as they did not always make it to the top 3.

*Yeah or end up in the top three on the leaderboard. It could maybe be a top ten list. So, you could see roughly where you are—or yeah, if you’re not even in the top ten, then depending on how many individuals are playing, maybe you’d feel like making an effort, or not?*

[67-year-old man]

Socializing and creating a community around exergaming was a nondigital reward mentioned during the interviews. Some participants who met during the introduction session reported that they took the initiative to organize group lunch meetings to discuss the game and walk together.

I *think it has been very fun and therefore I think that the other ladies will also continue exergaming, and then I thought we could maybe go for a walk. We can walk, and after we can go and have a coffee, and the ones who can’t walk that fast can rest more often*.

[71-year-old woman]

### Psychological Change Techniques

#### Overview

These techniques in the exergame aim to change the psychological outcomes of the users in real life. According to the framework, psychological change techniques include providing information, self-monitoring, and stress management. Even though the exergame was not designed to use the technique to provide information, participants experienced different levels of understanding of the exergame based on the information they received from the research team. Self-monitoring was used by the exergame and was thus mentioned by the participants. Stress management was not used in the design of the exergame; however, participants still mentioned stress and pain relief from getting immersed in the exergame. The gamification elements used in the exergame can be mapped to the techniques according to Table 3.

**Table 3.** The psychological change techniques experienced by people with chronic conditions after playing the Heart Farming walking–based exergame for 3 months and the gamification elements facilitating the techniques based on the deductive analysis using the gamification framework by Berglund et al [8].

Psychological change techniques as described in the framework	Heart Farming gamification elements
Provide information	The exergame did not use this technique. However, different levels of familiarity with the exergame were reported as an important factor in the experience of the exergame.
Self-monitoring	*Levels of achievements or ranks* increased motivation to progress in the exergame.
Stress management	Although stress management was not used as a technique, participants reported experiencing stress relief when exergaming. Some factors (eg, exergaming in public) unintentionally created stress for the participants.

#### Provide Information

The exergame did not use a technique to provide any health information, nor was this mentioned by the participants. However, different levels of understanding of the exergame were reported as a factor affecting their exergame experience and are thus presented under this section. Participants with previous experience with games found the exergame to be intuitive and easy to get into, while participants who were less familiar with technology found it to be complicated and hard to understand. A participant referred to her old age as a possible reason for not understanding the exergame.

#### Self-Monitoring

Participants found enjoyment in making progress in the game. The different levels they could reach in the game served as motivation and a goal to reach when playing.

*I probably like these different levels the best, it has been the most fun to go after them*.

[77-year-old man]

Participants also liked receiving a trophy for reaching their daily goals. Participants mentioned sometimes reaching their daily goal 2 or 3 times.

*Yes, I remember, it has happened a few times maybe that I have gotten many of these flags like when I have walked far now and then, and then I feel “wow, that was many of those flags*.”

[80-year-old woman]

#### Stress Management

The exergame did not use a technique to aid in stress management. However, playing the exergame could serve as stress relief, and specifically, an 80-year-old woman mentioned that *“it’s a relaxation at the same time that I need it.”* Participants highlighted the role of exergaming in relieving pain, as immersion in the exergame made it easier to distract from pain.

*I absolutely feel like it has only felt good, and then it can also be good if you have a day with a lot of pain, then you also forget a little, when you are like busy with these animals […] then I actually forget the rest a bit*.

[77-year-old man]

Participants also reported factors that unintentionally led to increased stress. Sometimes they thought that the game did not accurately measure how far they walked or that the exergame did not react to finger presses. Some worries were also expressed about what other individuals thought when they were exergaming in public.

*So, I have to walk like this, and then I have gotten the question* “*are you recording me?” from individuals nearby*.

[68-year-old woman]

## Discussion

### Main Contributions

This study aimed to explore the experiences of behavioral and psychological change techniques used by a walking-based exergame. Principal findings indicated that exergaming provided motivation to walk by using goals and rewards, and participants found value not only by progressing and getting rewarded in the exergame but also through experiences of getting out in nature or finding community with others. Participants found that exergaming assisted them in planning and structuring their daily routines, often playing in the mornings and integrating it with activities, such as visiting the doctor or grocery shopping. Several challenges were mentioned, including holding the phone and handling the camera or playing in public. Participants appreciated having support from the research team when being introduced to the exergame. Health-related impairments, such as knee problems, influenced the experience of exergaming. However, exergaming could also provide a distraction from pain.

Individuals’ capacities can influence their experience with exergaming, which includes their attitude toward exergaming, exergaming skills, and health-related impairments [27]. This was emphasized by the participants in our study, as they believed that their old age might have affected their capacity for exergaming. Previous qualitative studies indicated that exergaming can be experienced as a fun, engaging, and enjoyable activity for individuals with chronic conditions [13,39,40]. Participants in our study also enjoyed exergaming, although some were not as interested in exergaming and reported limited experiences with games prior to participating in this study. For an exergame to be experienced as an enjoyable activity, the skills and previous knowledge of participants should be evaluated [41]. Participants’ level of knowledge and skills with technology should be taken into consideration, since familiarization with technology can differ greatly for older adults [41]. For example, individuals may worry about their ability to play if they find the technology to be very advanced [14,41,42]. To overcome this challenge, individuals in our study received a structured familiarization with the exergame, including an introductory session and follow-up calls from the members of the research team who aimed to provide sufficient support, instruction, and feedback. Similar findings have been reported in other studies, highlighting the importance of familiarization when introducing exergames to older adults [27].

Social connection is a strong motivator for physical activity, especially in older adults [42-45], and could provide motivation to increase physical activity [41]. The exergame in our study included some support for social interaction within the exergame, where players could send emojis and crops to each other. However, participants in our study did not use the social functions in the exergame due to a lack of interest, difficulty understanding these functions, or not having anyone to play with. A previous study found a positive correlation between the number of messages players send to each other and their physical activity, highlighting the potential of social interaction within an exergame [46].

Participants in our study created social groups outside of the exergame where they walked together using the exergame but did not use the social interaction within the exergame. In another study, they also found that observing each other while exergaming can motivate players to play, even when playing single-player exergames [40]. Culture could also have an impact on how socialization affects exergame opportunities, as individualistic cultures are less likely to feel embarrassed, while older adults in other countries might experience discomfort from feeling judged by others [27]. In our study, there were also participants who stated that they are not interested in playing with others, as well as participants who expressed discomfort from playing the exergame in public, where other individuals could see them. This should also be considered when designing an exergame.

The participants of our study emphasized that exergaming is easy to incorporate into their daily lives, and they could combine daily activities, such as grocery shopping or doctor visits, with exergaming. In addition to walking 10 minutes more each day, study participants also mentioned that they integrated their daily activities with exergaming. Creating new routines is associated with increased adherence to exergaming [13]. To avoid interrupting their daily activities by stopping to play on the phone, participants of our study preferred to complete all their planning before they started walking and to handle all their rewards at the end of the walk. This indicates a preference for separating exercise from gameplay, which other exergames for older adults have utilized before [47].

Having the opportunity to exergame outdoors was considered an asset by the participants in our study. Exergaming motivated participants to go outside and engage with nature, exploring their surroundings instead of remaining inactive at home. However, individuals whose hands were unsteady or shaking while holding the phone could cause inaccurate measurements. Although participants in our study were offered armbands that could hold their phones without blocking the camera, this was not seen as ideal, and participants expressed a desire for another solution that would allow them to keep the phone in their pocket while walking, something that was not possible, as the technology used requires the phone’s camera. The alternative solution of using GPS instead introduces the issue of imprecise tracking in indoor environments, especially when walking short distances [16]. As participants used the exergame indoors under certain circumstances, for example, during bad weather, the ability to do so should still be considered when deciding what technology to use for this type of exergame.

Health-related impairments can impact individuals’ experience of exergaming [27]. The participants in our study were diagnosed with different chronic conditions (including stroke, chronic heart failure, Parkinson disease, and rheumatic disease) and varied in terms of symptom severity and disease progression. In our study, exergaming was considered an effective distraction from pain. This finding aligns with previous studies that have highlighted the significant role of exergaming in distracting from physical exertion and everyday life [10,27,38]. An RCT that compared brisk walking with exergaming in older adults also indicated that exergaming produced a lower level of perceived exertion, possibly due to distractions provided through exergame graphics or sound effects [47].

The results of our study show that future exergame design for individuals with chronic conditions should prioritize accessibility, adaptability, and meaningful integration into daily life. A systematic review by Cugusi et al [22] shows that exergames combining physical activity with cognitive engagement can enhance physical, cognitive, and mental health outcomes in older adults and people with chronic diseases while also supporting motivation and adherence. Tailored exergaming solutions that can be played at home, with adjustable difficulty levels and clear, intuitive feedback, are particularly relevant for addressing mobility, balance, and long-term engagement needs in clinical populations such as those with heart failure or dementia [30,48]. In line with these findings, it is essential to assess individuals’ skills and prior experience before developing or implementing exergames. Designing exergames that are safe, person-centered, and adaptable to varying functional abilities is crucial for supporting sustained participation among individuals living with chronic conditions [34].

### Strengths and Limitations

The strengths and limitations of this study ought to be taken into consideration when interpreting the results. The target sample included individuals with diverse chronic conditions, which differ in terms of symptom severity and illness progression. This provides a deeper understanding of the topic under investigation. In terms of transferability, the results may mirror the Swedish older population with certain chronic conditions, but there may be cultural differences, age differences, and differences if the game were to be used in other chronic conditions. Future studies are required to determine whether condition-specific exergames should be developed.

The use of this gamification framework assisted in structuring the analysis of the data. However, its use might have led to the absence of some important aspects discussed during the interviews. A few parts of the framework were not confirmed by the data, including the focus on past success, nonspecific rewards, threats, and self-monitoring as behavioral techniques. Although this framework underpinned the development of the Heart Farming exergame, it can be considered a broad, overarching model for game design. Therefore, it is understandable that not all the techniques described in this framework are employed in the Heart Farming exergame. It is also important to consider that the interviews were conducted by one of the game developers, which might have introduced potential biases. However, the data analysis was conducted by researchers with diverse academic backgrounds and clinical experience, which may have strengthened the trustworthiness of the findings.

While participants made promising statements regarding the effect the exergame had on their health, this study did not collect or analyze any potential health effects. To draw any conclusions about the actual health benefits of this type of exergame, future studies should include quantitative data on measurable health indicators to analyze how they are affected by exergaming.

### Conclusions and Implications

This study contributes to the exergaming literature by examining behavioral and psychological change techniques from the perspective of individuals with chronic conditions. To our knowledge, this is the first qualitative study to apply this gamification framework within this population. Considering the results of this study in combination with previous findings, exergaming can be a promising approach to improving physical activity and achieving behavioral change in individuals with chronic conditions. Considering individuals’ needs, previous knowledge of exergaming, interests, and illness progression is critical when developing and using exergames in health care. By increasing understanding of how game elements are experienced and how they influence health-related behavior, the findings of this study may inform the development of exergames that are better tailored to users’ needs.

Looking ahead, future studies with more diverse populations and larger sample sizes should focus on the evaluation of the impact of behavior-change techniques used in exergames to maximize the positive effects of exergaming in individuals with chronic conditions. It would also be valuable to explore whether different behavior-change techniques are required depending on the symptoms of diverse chronic conditions.

## Supplementary material

10.2196/78776 Multimedia Appendix 1 Interview guide used in the study.

## Abbreviations

Heart-eXg: Heart-eXergame
HF-CC: Heart Farming Chronic Conditions
RCT: randomized controlled trial
