# Supplementary material for: User Experiences of Behavioral and Psychological Change Techniques in a Walking-Based Mobile Exergame: Cross-Sectional Qualitative Study
Source: JMIR Serious Games. 2026 Mar 27;14:e78776. doi: 10.2196/78776 (PMC13027676; doi:10.2196/78776)
Supplement: Multimedia Appendix 1 [file games-v14-e78776-s001.docx]

**Interview Guide**

Thank you for choosing to participate in this interview. The questions in the interview will be about your participation in the study and your use of the Heart Farming game. I will ask questions about your experience of playing the game and the influence of the game on your daily life and habits. Afterward, I will focus on questions about the different elements of the game and their relation to your physical activity.

You can choose to end the interview at any point.

Shall we begin?

*Experiences of playing the game…*

1. Could you tell me about your experience of playing the game for the last 3 months?
2. Can you describe an ordinary day when you use the game?
   1. What time of the day do you use the game?
      1. Why do you choose to play at this specific hour?
      2. What impact do the reminders have on when you play the game?
   2. What do you usually do right before or directly after having used the game?
   3. In which environment do you use the game? Indoors or outdoors? Why?
   4. Did you usually use the game on your own or with others?
      1. If others, who did you choose to play the game with? How did you play together?
      2. If alone, what were the reasons for playing alone?
   5. What motivated you to play the game?

*The influence of the game in your daily life and habits…*

1. Do you think that the game influenced your life in any way? In what way/why not?
   1. Do you think that the game influenced your physical activity? In what way/why not?
   2. Do you think that the game influenced your daily habits? In what way/why not?
2. In what way do you think that your health has affected you playing the game?
   1. Did you face any challenges concerning your health when playing the game? If yes, please provide examples.

*Now some questions about the different elements of the game…*

1. How has using the game worked for you?
   1. Have you been able to use the game to play where (and when) you want to? If not, in what way did the game not allow you to play where you wanted?
   2. Have you felt unsafe or had any safety concerns when playing? If yes, please specify when and why.
   3. How easy would you say it is to understand and get into the game?
2. How has your understanding of the game been affected by the introduction and instructions of the game?
   1. Did you need help? If yes, with what? What did you do when you needed the help?
   2. Is there a need for more help? If yes, what kind of help and about what?
3. Can you describe how you usually play the game? (For example, do you have a special strategy, is there anything you tend to focus more on, do you make long or short plans…)
   1. Has the game allowed you to play the way you wanted? If not, how do you want to play and why do you feel you can’t play that way?
   2. What do you think about selling crops to neighbors? Unlocking decorations?
   3. What do you think about finishing missions to unlock animals?
   4. What do you think about the option to use autoplay?
      1. Do you use the function? If yes, when and why do you choose to do so?
         1. **If only/mainly using autoplay**: What makes you prefer autoplay over the other features?
         2. What do you think of the way autoplay works?
            1. Would you change anything about autoplay? What and why?
4. What do you think about the trophies for the daily goals?
   1. How do you feel when you receive them? When you don’t?
   2. Which impact did they have on your physical activity?
   3. Do you think three steps of rewards were enough? Did you want fewer/more?
5. What is your experience of challenges in the game? For example, game elements that challenge you to be more active or challenge you to think more.
   1. What do you think about the level of the challenges? Are they at an appropriate level? Why/why not?
6. What are your thoughts on the physical movements used to play the game?
   1. How do you feel about walking to do actions in the game? Why?
      1. If walking is a bad fit, what types of movements would you prefer?
   2. Do you do squats in the game?
      1. If not, can you explain why you don’t use the activity?
      2. If yes, what do you think about that activity?
   3. Would you like other physical movements in the game? Which ones and why?
7. What is your experience of playing together with other people?
   1. What is your experience of sending/receiving letters and crops from other people?
   2. What is your experience of the high score list for players?
   3. What is your experience of walking and playing together with someone else?
   4. How would you like to play with other people?
8. What do you think about the look of the game? (including animations)
9. What do you think about the sound effects?
10. What do you think of the game’s theme and environment, i.e., farming and the farm itself?
11. What do you like most about the game? Why?
12. What do you dislike most about the game? Why?
13. What would you like to change, remove, or add to the game? Why?
14. Would you recommend this game to anyone? Why/why not? (and who?)
    1. Is there a group of people you think this game is fitting for? Why/why not? (and who?)
    2. Would you like to continue using the game? Why/why not?

*Before wrapping up, I have a couple of questions about using games in general, not specifically Heart Farming, to be physically active.*

1. Do you think games can be used to increase physical activity? Explain
   1. Can games be used for other types of activities, such as specific exercises?
2. If we make other games like this in the future, what should we consider?

*That was the last of my questions. Is there anything else you would like to add before ending this interview?*

*Thank you for your participation in this interview.*
